# Real world experience of carglumic acid for methylmalonic and propionic acidurias: the second interim analysis of the multicentre observational PROTECT study

**DOI:** 10.1186/s13023-026-04522-z

**Published:** 2026-08-03

**Authors:** Sufin Yap, Elena Procopio, Alessandro Rossi, Alberto Burlina, Lucy Dougherty-de Miguel, Elvira Cañedo, Francesca Furlan, Sinziana Stanescu, Thibault Delcroix, Zahir Boutoub, Vincenzo Giordano

**Affiliations:** 1 Department of Inherited Metabolic Diseases, Sheffield Children’s Hospital, Sheffield, UK; 2 Metabolic and Muscular Unit, Department of Neuroscience, Meyer Children’s Hospital IRCCS, Florence, Italy; 3 Department of Translational Medicine, University of Naples “Federico II”, Naples, Italy; 4 Consultant Division of Inherited Metabolic Diseases, Reference Centre Expanded Newborn Screening, University Hospital Padova, Padova, Italy; 5 Pediatric Neurology, Vall d’Hebron University Hospital, Barcelona, Spain; 6 Department of Pediatrics, Universitat Autonoma de Barcelona, Bellaterra, 08193 Spain; 7 Department of Paediatric Nutrition and Metabolic Diseases, Hospital Universitario, La Paz, Paseo de la Castellana, 261, Madrid, 28046 Spain; 8 Fondazione IRCCS Ca’ Granda Ospedale Maggiore Policlinico, Regional Clinical Center for Expanded Newborn Screening, Milan, Italy; 9 Department of Pediatrics, Metabolic Disease Unit, Hospital Universitario Ramon y Cajal, University of Alcala de Henares, Madrid, Spain; 10 Recordati Rare Diseases SARL, Puteaux, France

**Keywords:** Propionic aciduria, Methylmalonic aciduria, Carglumic acid (Carbaglu^®^), Long-term treatment, Hyperammonaemia

## Abstract

**Introduction:**

Propionic aciduria (PA) and methylmalonic aciduria (MMA) are rare inherited metabolic disorders marked by recurrent metabolic decompensation and hyperammonaemia, resulting in neurological morbidity, chronic complications, and substantial healthcare burden. Although carglumic acid (Carbaglu®) is established for acute hyperammonaemic crises, evidence for its long-term use is limited. This study reports the 18-month interim analysis of PROTECT, a large prospective observational study evaluating chronic Carbaglu® therapy in PA and MMA.

**Methods:**

PROTECT is a prospective, longitudinal, observational study conducted across 34 centres in seven European countries. Patients with PA or MMA receiving long-term Carbaglu® for at least six months were eligible. Clinical data were extracted from medical records and analysed descriptively. Annualised pre- and post-treatment comparisons were performed in patients with at least six months of life without long-term Carbaglu® prior to initiation. Outcomes included metabolic decompensation events, ammonia control, healthcare utilisation, and growth parameters.

**Results:**

Fifty-eight patients were included in this interim analysis. Among 39 evaluable patients, long-term Carbaglu® treatment was associated with reduced annualised metabolic decompensation events, including hyperammonaemic episodes. Mean peak ammonia levels during decompensations and background ammonia concentrations were significantly lower following treatment initiation. Healthcare utilisation decreased substantially, with significant reductions in inpatient admissions, inpatient days, and emergency room visits. Growth parameters remained stable in patients initiating treatment later in life, while patients treated from birth demonstrated sustained metabolic stability and favourable growth profiles.

**Discussion:**

Despite heterogeneity and limitations inherent to real-world observational data, these findings indicate clinically meaningful improvements in metabolic control and healthcare burden with long-term Carbaglu® therapy. Reductions in both acute and background ammonia exposure suggest potential cumulative neuroprotective benefits.

**Conclusions:**

This 18-month interim analysis supports Carbaglu® as a beneficial long-term treatment option for patients with PA and MMA, with particular promise when initiated early in life. Ongoing follow-up will further clarify long-term outcomes and inform optimisation of chronic management strategies.

**Clinical trial statement:**

Findings based on clinical trial NCT04176523 titled the PRospective Observational study of long-TErm Carbaglu® for the Treatment of PA & MMA (PROTECT), registered retrospectively at ClinicalTrials.gov on 25th November 2019, available at https://clinicaltrials.gov/study/NCT04176523.

## Introduction

Propionic aciduria (PA) and methylmalonic aciduria (MMA) are organic acidurias resulting from propionate metabolic pathway defects. PA arises due to mutations encoding the mitochondrial enzyme propionyl-Coenzyme A (CoA) carboxylase, causing propionyl-CoA and associated byproducts 3-hydroxypropionic acid and 2-methylcitric acid to accumulate [1, 2]. MMA, a distinct but similar disease, arises from defects in methylmalonyl-CoA mutase or its cofactors, causing methylmalonyl-CoA and its byproducts to accumulate [3, 4]. The resultant CoA depletion and secondary cataplerosis associated with low blood glutamate and glutamine [5], causes deficiency of N-acetylglutamate (NAG) synthesis, essential to the urea cycle – resulting in hyperammonaemia [6]. In PA there is significant impact on the TCA cycle. The excess of propionyl-CoA leads to utilisation of oxaloacetate, an important component of this cycle. The resultant formation of methylcitrate deprives the cycle of citrate leading to a reduction in ATP production [5].

PA and MMA are usually detected in the first month of life, neonates typically present with metabolic acidosis, hyperammonaemia and clinical deterioration [4, 7]. Neonates affected must be diagnosed swiftly and stabilisation of the metabolic decompensations is time critical to avoid neurological complications, coma and death [4]. These metabolic decompensations can continue throughout life with potentially dangerous consequences [7].

Carglumic acid (Carbaglu^®^) which reactivates the urea cycle, has been indicated in treatment of hyperammonaemia due to MMA and PA [8] with many studies confirming its effectiveness in the acute treatment of hyperammonaemic decompensation events [9, 10]. Retrospective observational studies [10, 11] and case reports [12–15] show reduction in ammonia levels in neonates and older patients with OAs, irrespective of the use of scavenger medication, meaning Carbaglu^®^ is used without scavengers after confirmed diagnosis of OA.

PA and MMA cause chronic complications with poor physical and cognitive development, end-organ damage including progressive renal disease (mainly MMA) and cardiac diseases (mainly PA). MMA and PA long-term management goals are to minimise the number of metabolic decompensation events and achieve normal development.

The cornerstone of chronic therapy is a protein restricted diet. In the 2014 guidelines this was defined as a protein restricted diet with synthetic protein supplement (devoid of precursor amino acids), vitamin and mineral supplementation, L-carnitine at 100–200 mg/kg/day, antibiotics to reduce propionic acid production in the gut and vitamin B12 in responsive MMA patients [4]. Chronic therapy involves a multifaceted approach: dietary management; drug therapy; life-long monitoring to identify and treat potential complications [7]. In patients with brittle biochemical control despite chronic therapy, liver transplantation or combined liver-kidney transplantation may be beneficial [16]. Recent evidence suggests that early liver transplantation improves metabolic stability and can reduce the risk of long-term complications [17]. Transplantation in patients with PA and MMA is associated with significant risks, including a 14–22% risk of post-transplant mortality, a 19% risk of graft failure or rejection and the persistence of disease-related complications despite transplantation [16–18]. Whilst survival is improving, with many OA patients living into adulthood, the life expectancy and quality of life of MMA and PA patients is significantly less than that of the general population [19].

Recently, several studies have been published concerning the impact of long-term Carbaglu^®^ treatment on PA and MMA patients. It reduces the number of decompensation events and/or hospitalisations [20–26]; reduces mean plasma ammonia [20, 21, 23, 26, 27] and allows optimised natural protein intake [20, 21, 24, 27]. Other secondary benefits reported include improved growth and cognition [20]. The cumulative reduction in background ammonia levels due to NAG substitution with Carbaglu^®^ and the consequent better functioning urea cycle can explain improvements in cognition [28]. Meanwhile, growth is energy-requiring, and the urea cycle has an anaplerotic effect. When the urea cycle is functioning, fumarate is produced, helping to replenish the TCA cycle and maintaining ATP production.

This paper presents the second (18-month) interim analysis of the **PR**ospective **O**bservational study of long-**TE**rm **C**arbaglu^®^ for the Treatment of PA & MMA (PROTECT). PROTECT is the largest long-term study of Carbaglu^®^ in patients with PA and MMA and was registered retrospectively at ClinicalTrials.gov (NCT04176523) on 25th November 2019. It builds upon the findings of the first PROTECT interim (12 month) analysis previously published [26].

## Methods

### Study design and participants

PROTECT is a prospective, longitudinal, observational study of patients diagnosed with MMA or PA. The observational study population includes patients from 7 European countries: France, Italy, the UK, Spain, Sweden, Germany, and Norway. Enrolment is ongoing having begun in January 2019. All enrolled patients must have been genetically and / or biochemically diagnosed with PA or MMA and treated for a minimum of 6 months long-term treatment with Carbaglu^®^. All patients meeting this are eligible – there are no exclusion criteria.

The study is conducted in accordance with all local and national legal and regulatory requirements and with the 1964 Declaration of Helsinki, as revised in 2013. Prior to inclusion, written informed consent has been provided by all participants or caregivers as appropriate.

### Objectives

The primary and secondary study objectives included for analysis within this paper are outlined in Table 1.

**Table 1:** PROTECT study objectives

Objective Type	Metric	Data capture	Analysis
Primary	Hyperammonaemic decompensation events*	Number and duration of events, ammonia levels	Annualised data, pre-Carbaglu^®^ treatment initiation vs. post-Carbaglu^®^ treatment initiation
Secondary	Background Ammonia Level	Plasma ammonia levels outside decompensation events	Annualised data, pre-Carbaglu^®^ treatment initiation vs. post-Carbaglu^®^ treatment initiation
Secondary	Health-service Burden	In-patient events, in-patient days, ER visits	Annualised data, pre-Carbaglu^®^ treatment initiation vs. post-Carbaglu^®^ treatment initiation
Secondary	Growth	Weight and height	Annualised data, pre-Carbaglu^®^ treatment initiation vs. post-Carbaglu^®^ treatment initiation
Secondary	Treatment with long-term Carbaglu^®^ from birth	Impact on all above metrics when treatment started from birth	Observational cohort analysis

* Hyperammonaemic decompensation events are defined as an increased ammonia level (> 159 µmol/L during the first month of life or > 60 µmol/L after 1 month of age), with an increased lactate level (> 1.8 mmol/L) and/or acidosis (pH < 7.35) [26]

### Data collection

**Fig. 1:**
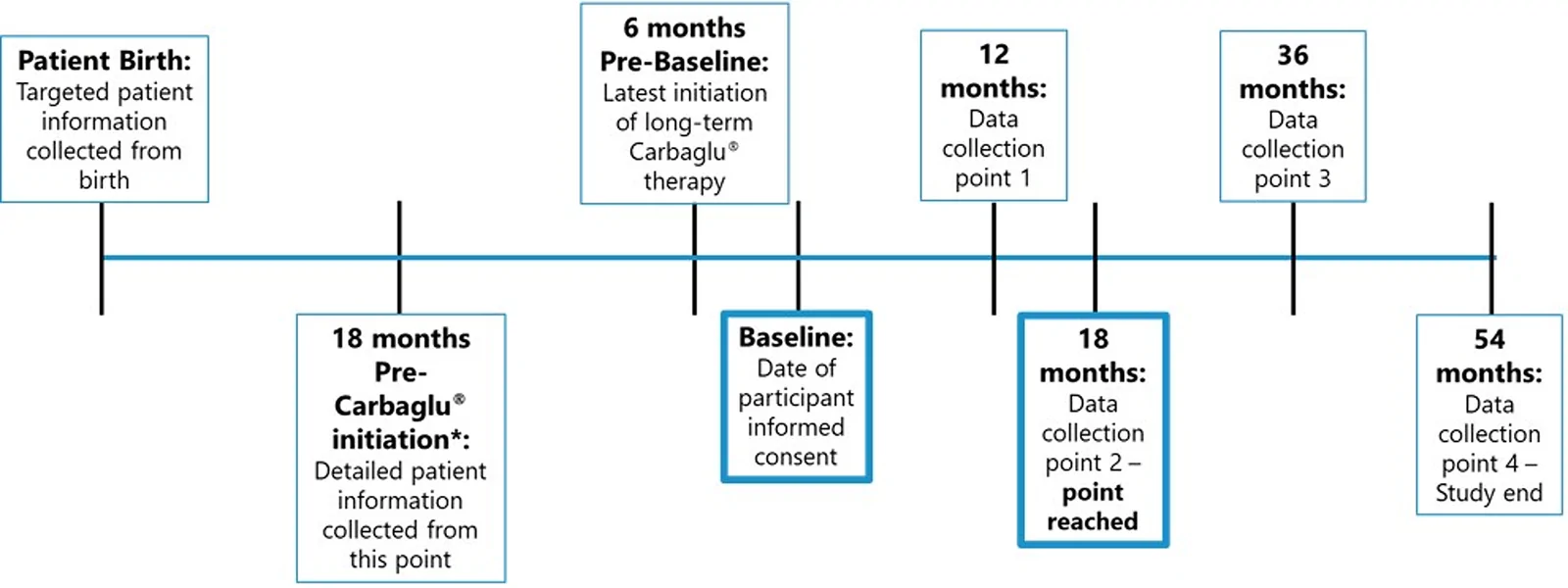
Data collection overview PROTECT study

Data are collected from the patient record, and an electronic data capture platform is utilised to capture and store all data. Data captured addresses the study objectives. Figure 1 outlines the data collection strategy utilised in the PROTECT study, for those patients who experienced at least 6 months of life without long-term Carbaglu^®^ treatment. The study has reached the second data collection point timed at 18 months.

### Data analysis

Patient data pre and post initiation of long-term Carbaglu^®^ treatment was annualised per patient, aggregated per metric, and compared. Within our analysis it was stipulated only patients who had experienced at least 6 months of life without long-term Carbaglu^®^ treatment could be included in annualised pre and post treatment initiation comparisons. Data from study participants treated from birth was analysed across the same metrics without having comparable pre-treatment data.

Data analysis utilised descriptive statistics. The mean, median and range, and absolute and relative frequencies summarised continuous and categorical variables respectively. Statistical significance was calculated across all comparable metrics at 95% confidence.

## Results

### Participant sample

There are 77 patients enrolled in the study, 58 of these were included in this analysis. 19 patients were excluded. Of those excluded, 16 patients discontinued in the study and 3 patients had unresolved data queries at the time of the analysis.

Of those included in the analysis, 39 of these participants experienced at least 6 months without long-term Carbaglu^®^ treatment pre-treatment initiation allowing the comparison of annualised 18-month pre-Carbaglu^®^ treatment data with annualised data from long-term Carbaglu^®^ treatment initiation to 18-months post-baseline. 19 patients started long-term Carbaglu^®^ treatment within 6 months of birth and have insufficient data for pre-treatment comparison. Table 2 summarises the characteristics of the participant sample.

**Table 2:** Participant sample characteristics

Sex	Male	28
	Female	30
Diagnosis	PA	40
	MMA	18
Baseline age (months)	Mean	105
	Range	6–413
Carbaglu^®^ Dosage* (mg/kg/d) (*n* = 50)	Mean	40
	Range	5–118

*Dosage determined by treating physician, mean calculated based on latest maintenance treatment dose per patient. Specific Carbaglu^®^ adherence data not recorded

### All decompensation events (Fig. 2)

**Fig. 2:**
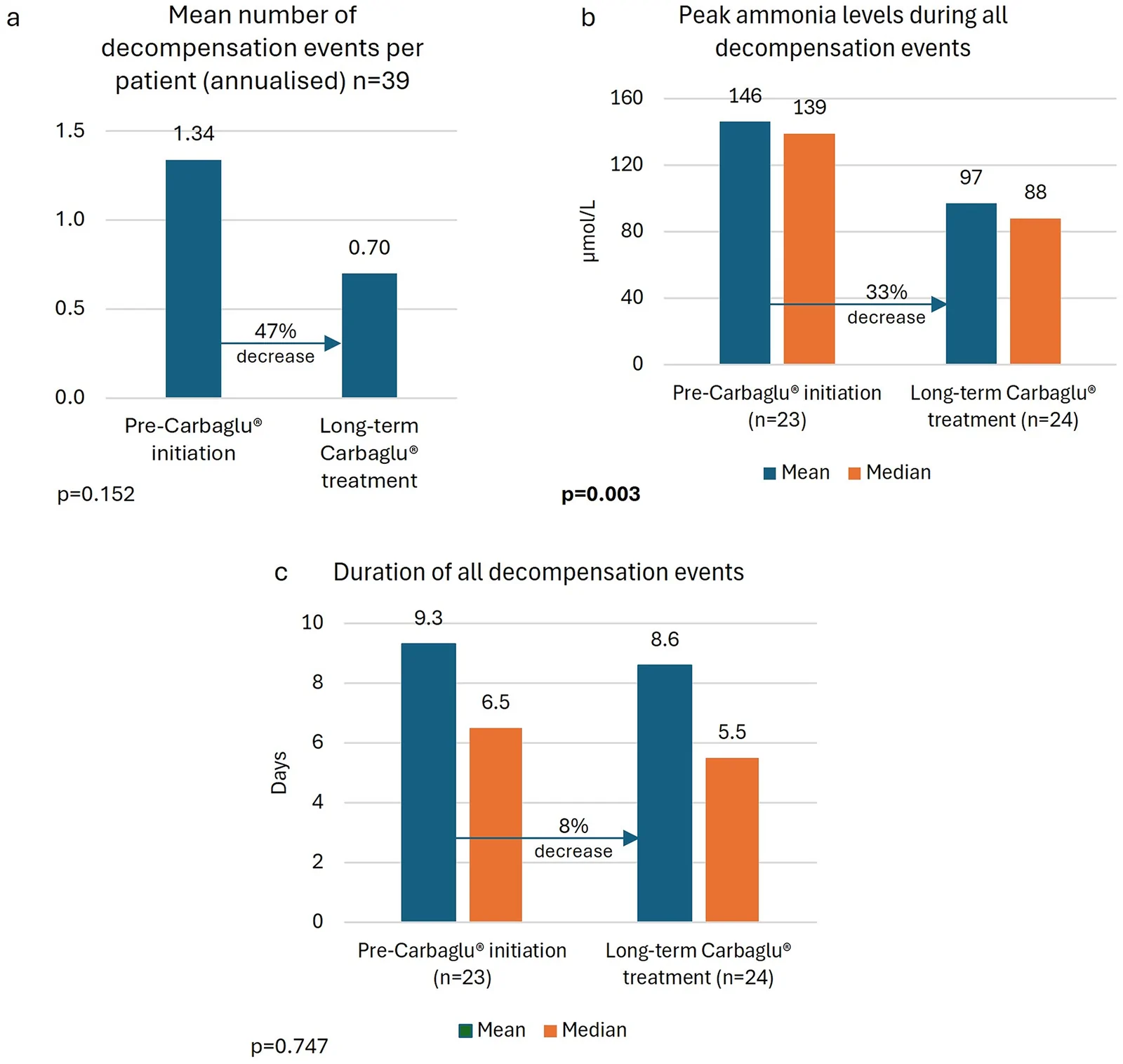
Effect of long-term Carbaglu^®^ treatment on all decompensation events. Bar graphs show (**a**) mean number of decompensation events per patient per year, (**b**) peak plasma ammonia levels during decompensation events, and (**c**) mean decompensation event duration, before and after treatment initiation. A statistically significant improvement was observed for peak ammonia levels (*p* = 0.003), whereas reductions in event frequency and duration did not reach statistical significance (*p* = 0.152 and *p* = 0.747, respectively)

Across all decompensation events the mean (SD) number of decompensation events per patient per year decreased from 1.34 (2.42) pre-treatment initiation to 0.70 (1.34) post long-term treatment initiation across the 39 evaluable patients (Fig. 2a). This was a non-significant 47% reduction in mean *p* = 0.152.

When treated with Carbaglu^®^ 44% of participants saw a decrease in number of decompensation events per year, 28% saw an increase in decompensation events per year and 28% saw no change. All those with no change in the number of decompensation events experienced zero decompensation events before and after treatment initiation (data not shown).

Mean (SD) and median peak ammonia levels across all decompensation events (Fig. 2b) decreased from 146 (63) µmol/L and 139 µmol/L respectively pre-treatment initiation where *n* = 23, to a mean (SD) of 97 (41) µmol/L and median of 88 µmol/L post treatment initiation where *n* = 24. The 33% decrease in mean was statistically significant *p* = 0.003.

Figure 2c shows Mean (SD) and median duration of all decompensation events pre-treatment initiation equalled 9.3 (8.5) days and 6.5 days respectively (*n* = 23). Post treatment initiation mean (SD) duration of decompensation events was 8.6 (6.9) days and median duration was 5.5 days (*n* = 24). The 8% decrease in mean duration of decompensation event was not statistically significant *p* = 0.747.

### Hyperammonaemic decompensation events (Fig. 3)

**Fig. 3:**
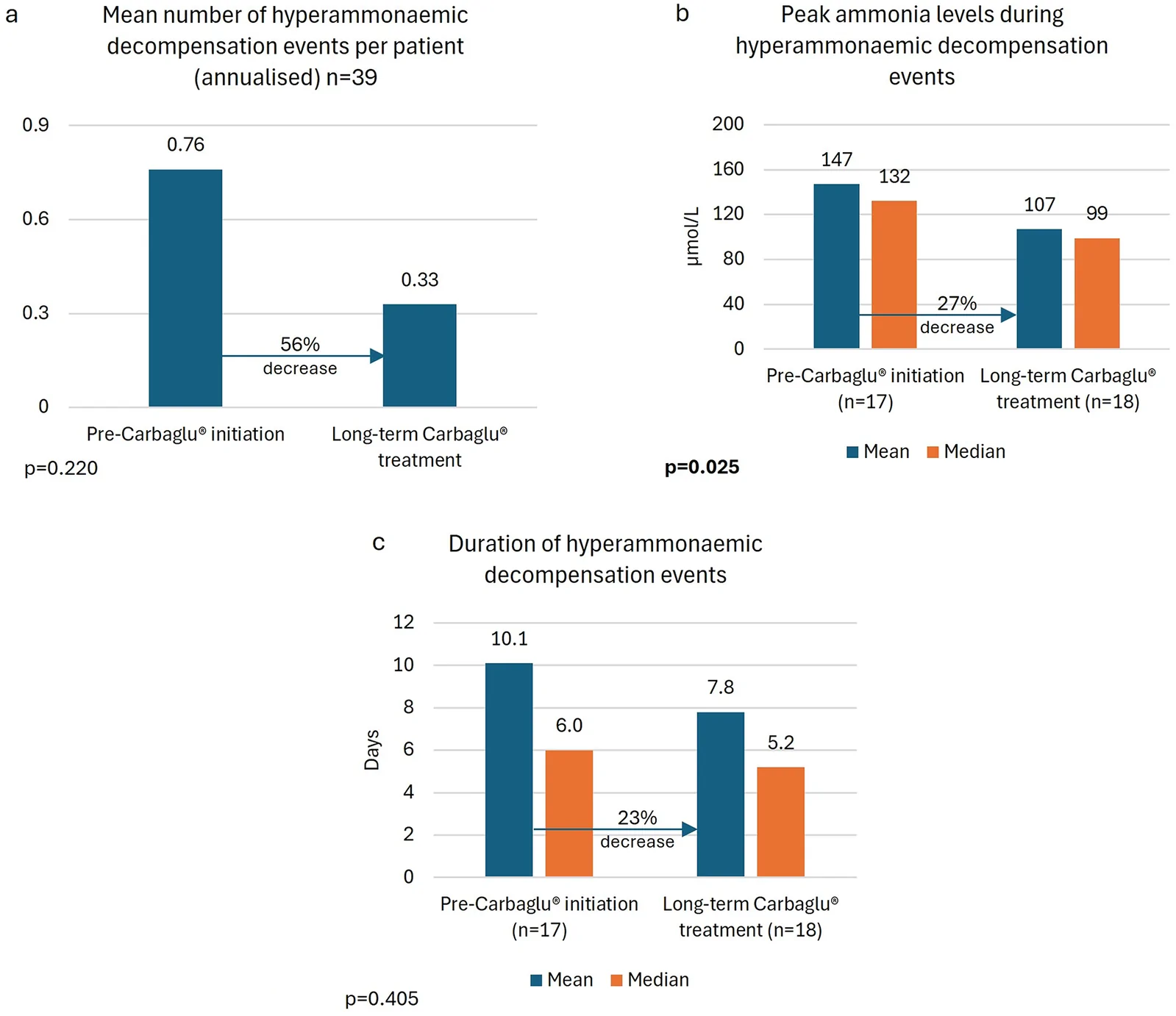
Changes in hyperammonaemic decompensation characteristics before and after long-term Carbaglu^®^ treatment. (**a**) Mean number of hyperammonaemic decompensation events per patient per year decreased following treatment (*p* = 0.220). (**b**) Peak plasma ammonia levels during hyperammonaemic episodes were significantly lower post-treatment (*p* = 0.025). (**c**) Duration of hyperammonaemic decompensation events showed a non-significant reduction after treatment (*p* = 0.405)

Hyperammonaemic decompensation events are defined as an increased ammonia level (> 159 µmol/L during the first month of life or > 60 µmol/L after 1 month of age), with an increased lactate level (> 1.8 mmol/L) and/or acidosis (pH < 7.35) [26]. Figure 3a shows the mean (SD) number of hyperammonaemic decompensation events per patient per year fell from 0.76 (1.98) pre-treatment initiation to 0.33 (0.97) post long-term treatment initiation (*n* = 39). This 56% decrease in hyperammonaemic decompensation events per year per patient after long-term treatment with Carbaglu^®^ was not statistically significant *p* = 0.220. When treated long-term with Carbaglu^®^ 36% of participants saw a decrease in number of hyperammonaemic decompensation events per year, 18% saw an increase and 46% saw no change. All those with no change did not experience a hyperammonaemic decompensation event (data not shown).

Mean (SD) and median peak ammonia levels during hyperammonaemic decompensation events pre-treatment initiation were 147 (67) µmol/L and 132 µmol/L respectively (*n* = 17) (Fig. 3b). Once long-term treatment with Carbaglu^®^ began the mean (SD) and median peak ammonia levels were 107 (27) µmol/L and 99 µmol/L (*n* = 18). This represents a 27% decrease in mean ammonia level, statistically significant *p* = 0.025.

Mean (SD) and median duration of hyperammonaemic decompensation events was 10.1 (9.9) days and 6.0 days pre-treatment initiation (*n* = 17). The mean decreased post-treatment long-term treatment initiation to 7.8 (5.5) days, while the median decreased to 5.2 days (*n* = 18) respectively (Fig. 3c). The 23% reduction in mean was not considered statistically significant *p* = 0.405.

### Background ammonia levels (Fig. 4)

**Fig. 4:**
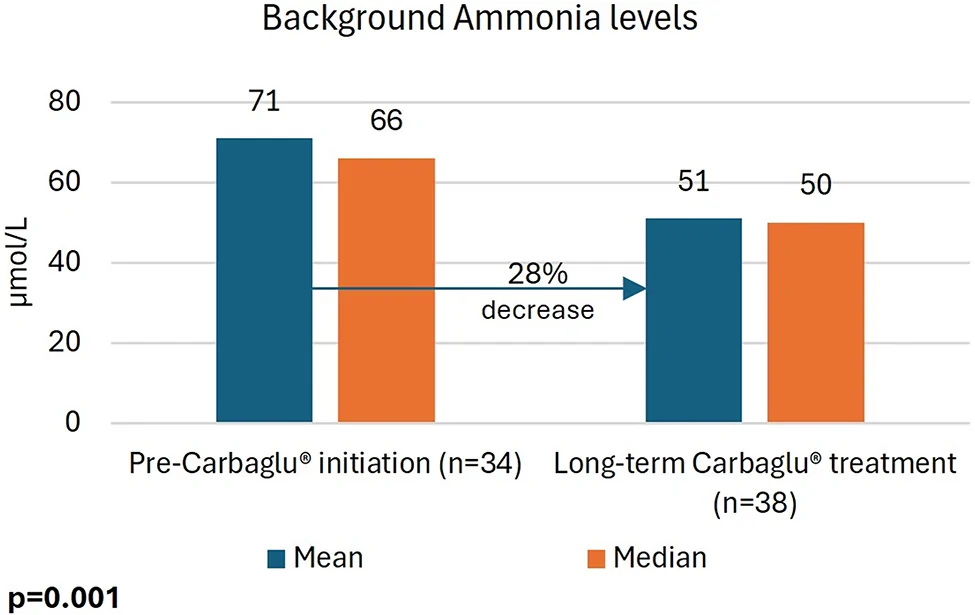
Background ammonia levels before and after long-term Carbaglu^®^ treatment. Background ammonia was defined as plasma ammonia measured outside of metabolic decompensation events. Mean background ammonia levels were significantly reduced following treatment (*p* = 0.001)

Background ammonia levels are considered as any blood plasma ammonia reading taken outside of a metabolic decompensation event. Figure 4 shows 34 patients had data concerning background ammonia levels prior to the initiation of long-term Carbaglu^®^ treatment, the mean (SD) and median background ammonia levels of this population was 71 (31) and 66 µmol/L. 38 patients had background ammonia level data post-treatment initiation, the mean (SD) was 51 (18) µmol/L and the median 50 µmol/L. The 28% decrease in mean was statistically significant *p* = 0.001.

### Health service burden (Fig. 5)

**Fig. 5:**
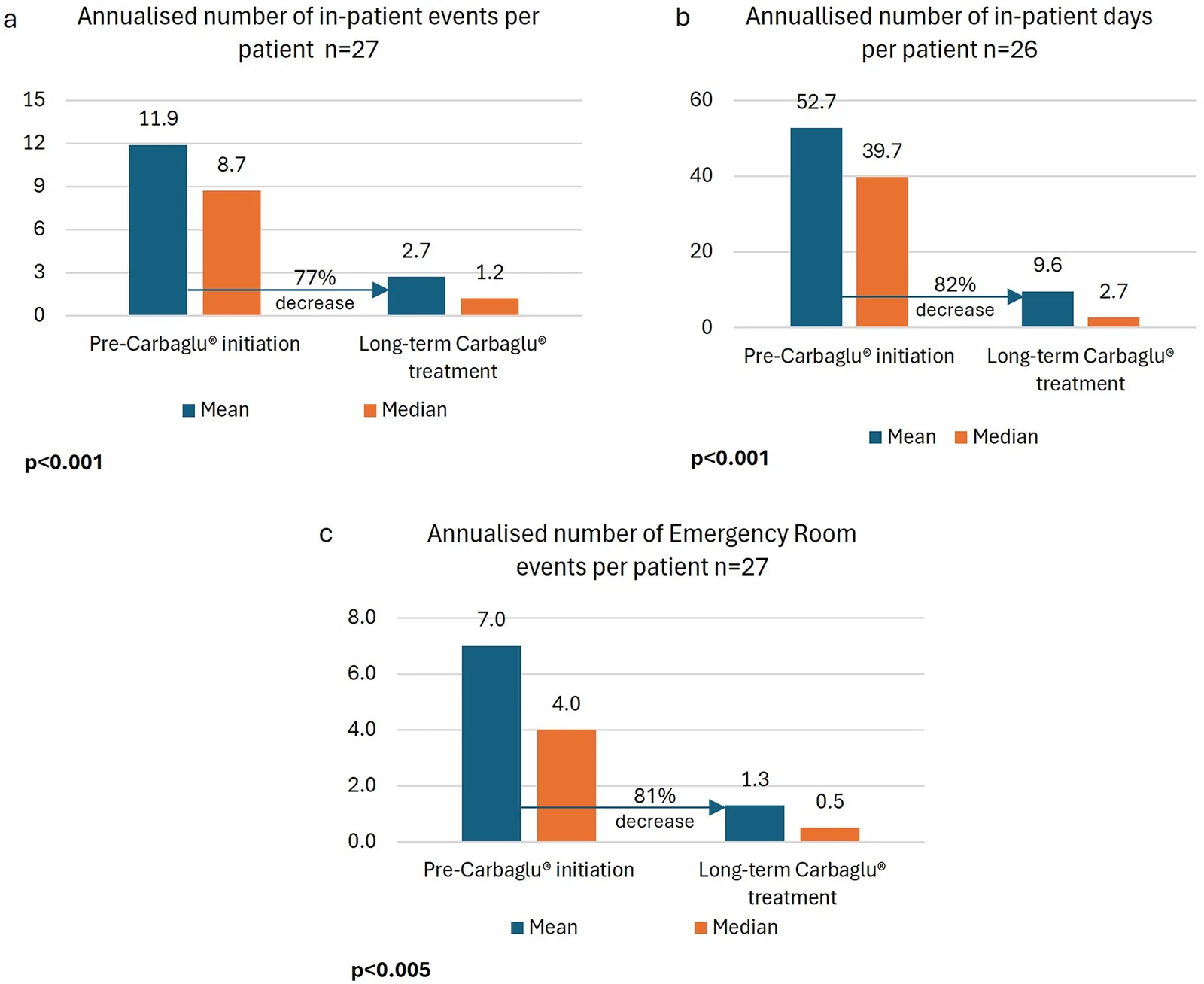
Reduction in healthcare utilization during long-term Carbaglu^®^ treatment. (**a**) In-patient events per patient per year, (**b**) in-patient days per patient per year, and (**c**) emergency room events per patient per year were all significantly reduced following treatment (*p* < 0.005)

Across all metrics, health-service burden was significantly reduced (*p* < 0.05) during long-term Carbaglu^®^ treatment compared with the pre-treatment period (Figs. 5a-c).

Mean (SD) number of in-patient events per patient per year decreased from 11.9 (11.2) days to 2.7 (3.7) days (*n* = 27), *p* < 0.001 for this 77% decrease.

Mean (SD) number of in-patient days per patient per year saw an 82% decrease across the 26 patients with comparable data, from 52.7 (40.0) pre-treatment to initiation to 9.6 (15.2) post treatment initiation, this change in mean was significant at *p* < 0.001.

The number of emergency room events per patient per year was comparable in 27 patients. The mean (SD) decreased 81% from 7.0 (7.9) pre-treatment initiation to 1.3 (1.8) once patients were treated long-term with Carbaglu^®^, this significant change in mean was calculated at *p* < 0.005.

### Patient weight and height (Fig. 6)

**Fig. 6:**
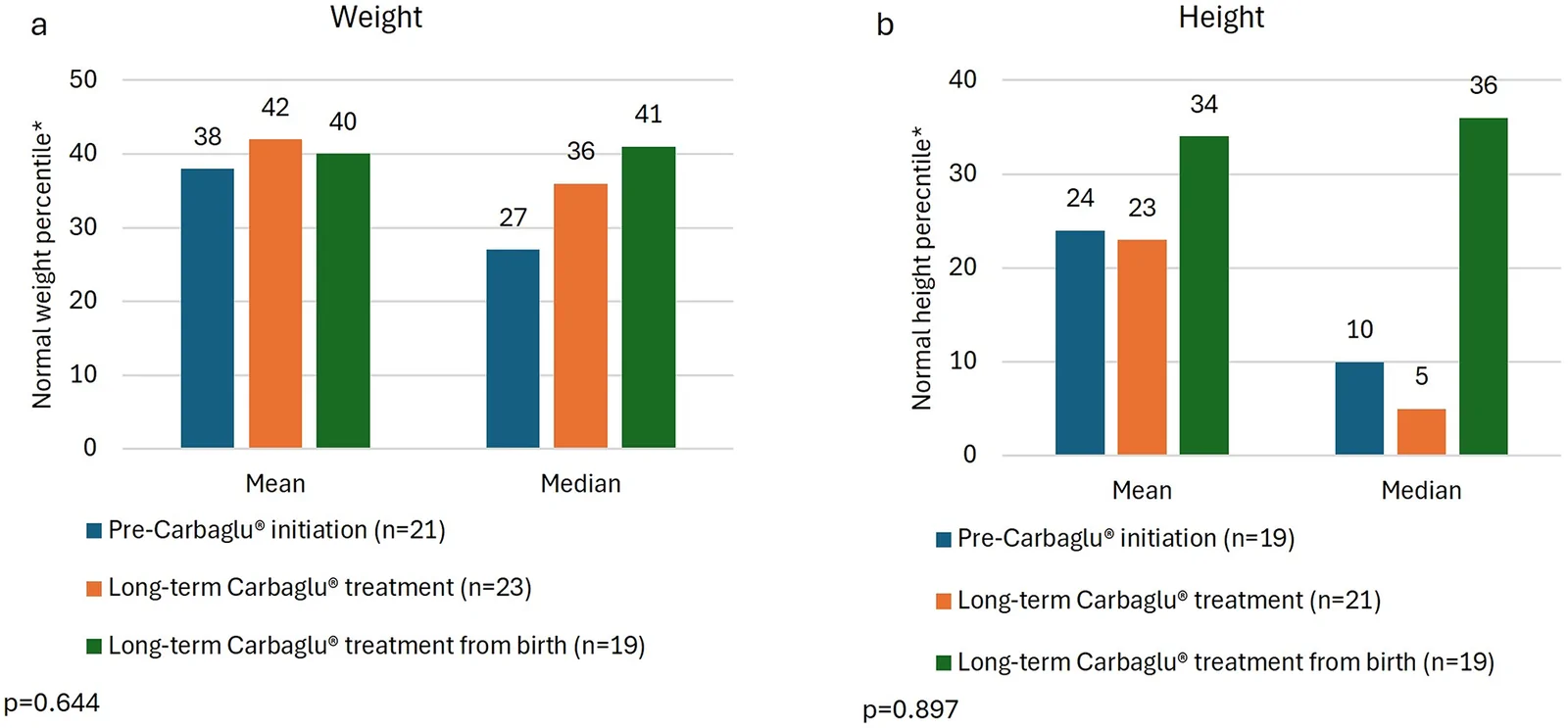
Growth outcomes during long-term Carbaglu^®^ treatment. (**a**) Weight percentiles and (**b**) height percentiles (CDC growth chart–normalized) showed no significant change following long-term treatment, based on mean percentile comparisons (weight: *p* = 0.644; height: *p* = 0.897) [29]. Growth outcomes for patients treated from birth are also displayed

Patient weight and height were compared to expected weight and height for age using CDC growth charts (https://www.cdc.gov/growthcharts/cdc-data-files.htm) (Fig. 6a, b) [29]. 21 patients between them had 63 weight readings taken pre-treatment initiation and 23 patients had 282 readings taken post long-term treatment initiation. Pre-treatment initiation mean (SD) normal weight percentile was 38 (34) and median 27. Following long-term treatment initiation mean (SD) normal weight percentile increased to 42 (28) and median to 36. There was a 12% increase in mean although not considered statistically significant, *p* = 0.644.

19 patients between them had 62 height readings taken pre-treatment initiation and 21 patients had 273 readings taken post-treatment initiation. Mean (SD) and median normal height percentile was 24 (28) and 10 respectively pre-treatment initiation. Post treatment initiation mean (SD) normal height percentile was 23 (28) and the median 5. The 5% decrease in mean height percentile was not statistically significant *p* = 0.897.

In patients who had been treated from birth mean (SD) and median weight percentile was 40 (23) and 41 respectively across 227 readings from 19 patients. Across these 19 patients treated from birth there were 192 readings for height, their mean (SD) and median normal height percentiles were 34 (27) and 36 respectively.

### Patients receiving long-term Carbaglu^®^ treatment since birth (Fig. 7)

**Fig. 7:**
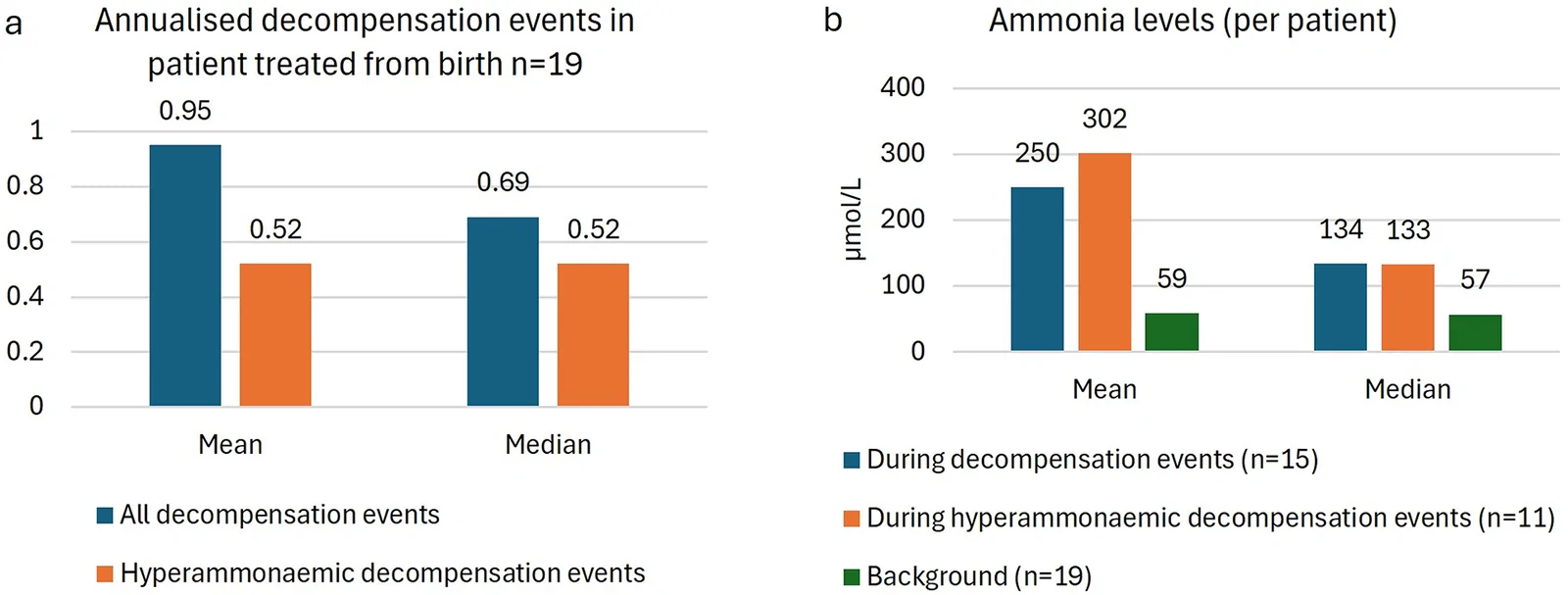
Clinical outcomes in patients who initiated long-term Carbaglu^®^ treatment within the first 6 months of life. (**a**) Annual rates of all metabolic and hyperammonaemic decompensation events while receiving treatment. (**b**) Peak plasma ammonia levels during decompensation events and background ammonia levels outside decompensation

Figure 7a shows the mean (SD) and median number of all metabolic decompensation events per patient per year in patients beginning long-term Carbaglu^®^ treatment within 6 months of life once receiving treatment was 0.95 (0.92) and 0.69 respectively (*n* = 19). The mean (SD) and median number of hyperammonaemic decompensation events per patient per year in patients beginning long-term Carbaglu^®^ treatment within 6 months of life once receiving treatment was 0.52 (0.57) and 0.52 respectively (*n* = 19).

Ammonia levels are demonstrated in Fig. 7b. Across all decompensation events mean (SD) and median peak ammonia levels were 250 (316) µmol/L and 134 µmol/L respectively (*n* = 15). Across these hyperammonaemic decompensation events mean (SD) and median peak ammonia levels were 302 (386) µmol/L and 133 µmol/L respectively (*n* = 11). Mean (SD) and median background ammonia levels across this 19-patient sample were 59 (17) µmol/L and 57 µmol/L respectively.

## Discussion

The PROTECT study is a prospective observational study and this interim analysis of 58 patients represents a large data source. Like any large real-world observational studies in rare diseases, any study is difficult to design and hard to enact as previously outlined in papers by Augustine 2013 and Mew 2018 [30, 31]. Studies in PA and MMA also have significant challenges as the clinical presentation of PA and MMA varies significantly even within families [32]. Heterogeneity is built into this type of study from a number of factors. These include but are not limited to:

- The accuracy and completeness of the patient record in the participating centres both prior to, and post study initiation.
- The accuracy and completeness of the data to the study eCRF.
- Many analyses omitted patients due to inaccuracies, omissions or ambiguities in data reporting.
- Due to the above some of the analyses have small sample sizes which can lead to insufficient statistical power.
- The study involved 34 sites across 7 countries, and each will have nuances in clinical practice. The study looks essentially at patients who are treated with the current standard of care by that practitioner in that centre, plus long-term Carbaglu^®^.
- The age of entry into the study is variable.
- The home management of patients will be varied depending on both advised medical practice and the understanding and adherence to it.
- Hospitalisations may be partially dependent on patient/ carer/ parent attitude, proximity to a hospital and:
  - The genetic determinant of the disease in each patient.
  - The impact of the management of concomitant complications.

This broad spectrum of heterogeneity may reduce the ability of the trial to demonstrate treatment effect [33]. Observational studies can also suffer from data collection issues, and these affected this analysis. Initially 60 subjects were identified as having reached the 18-month post baseline data collection point, 2 had to be removed from analysis regarding insufficient data, reducing the power of statistical analysis.

Furthermore, across all analysis completed we hoped to be able to factor in Carbaglu^®^ dosage, unfortunately inconsistencies in data reporting prevented this, it is being addressed ahead of the next analysis. Finally, when considering participant growth and height it is important to understand their caloric and protein intake, again insufficient data prevented the opportunity to include these metrics in the analysis. Again, we hope to have the necessary dataset accurately completed for the next analysis.

Despite these limitations, the data are significant and provides further insights into how to optimise the management of PA and MMA chronically.

### Decompensations and ammonia levels

The primary end point of the study was to describe the number and duration of hyperammonaemic decompensation events in patients with a diagnosis of MMA or PA who have been receiving Carbaglu^®^ for a minimum of six months compared to the number and duration of decompensation events that occurred prior to starting treatment with Carbaglu^®^.

While any decompensation event is a metabolic crisis that requires urgent treatment, the higher a patient’s ammonia levels the greater the risk of life-threatening neurological complications [34]. Therefore, any reduction in these events could be considered a clinically significant result.

The 18-month analysis shows a 56% decrease in hyperammonaemic decompensation events per year per patient after long-term treatment with Carbaglu^®^. The mean and median duration of the decompensation events associated with hyperammonaemia also decreased by 23% and 13% respectively. For the average patient therefore, we have two factors both contributing to a reduction in the toxic effect of ammonia. There is also a third. The results show us that the maximum levels of ammonia in hyperammonaemic decompensation events were reduced by 27% across the cohort analysed. This mean ammonia level reduction was statistically significant (*p* = 0.025) and the median fell by a corresponding 25%.

This reduction in decompensation events with hyperammonaemia, the reduction in the duration of these events and the reduction in maximum levels of ammonia achieved during these events represent a cumulative effect on ammonia that is of potential clinical value, as high levels of ammonia are associated with CNS neurological damage. Many clinicians suggest that it is not just the level of ammonia but the cumulative amount of hyperammonaemia that is responsible for its deleterious clinical effects, so this observation may have real implications on long-term outcomes.

Across all decompensation events (both hyperammonaemic and non-hyperammonaemic), a similar pattern is seen as in decompensation events associated with hyperammonaemia. There was a reduction in the mean number of decompensation events per patient per year of 47% for patients treated with long-term Carbaglu^®^. Although this reduction was reported as non-significant its p-value is 0.152 indicating reasonable confidence in the data. The duration of these events reduced by 8% in the long-term Carbaglu^®^ group with a statistically significant 33% reduction in peak ammonia levels. Again, the cumulative effect of these factors may have the most significance from a patient outcome perspective.

The authors have considered that there may be a synergistic impact based on all three factors namely decompensation events with hyperammonaemia, length of these events and peak ammonia levels. The concept is that the neurological damage caused by ammonia is a function of how high the ammonia is, how long it stays high and how often it happens. We can create a single index that combines these factors together to create a value that represents the total annual neurotoxic load. We have termed this the Cumulative Ammonia Burden Index (CABI). The CABI can be calculated from the formula; (Days of Hospital Admission / Year) x (Peak µmol/L) = CABI. This approximates to (Total Exposure Days / Year) x (Severity). It could be argued that peak ammonia should be calculated as peak levels of ammonia above the normal range as this is the truer measure of elevation although as this is an index it might not be important.

Based on the data presented and recognising that it comes from different data sets we can crudely calculate that the total time spent in decompensation events associated with hyperammonaemia dropped from 7.67 days/year pre-Carbaglu^®^ to 2.57 days/year after long-term treatment. This we could refer to as a reduction in “toxic state” of 66.5%. During the toxic state the CABI pre Carbaglu^®^ was 1127 compared to 275 for long-term Carbaglu^®^ – a reduction in the CABI of 75%.

The concept that the duration of hyperammonaemia and the peak level are both important predictors of neurological outcome is consistent with the literature. The “Consensus guidelines for management of hyperammonaemia in paediatric patients with urea cycle disorders” directly address this issue. It explicitly states, “two factors determine the prognosis of neurological damage: the duration of hyperammonaemic coma and plasma ammonia levels”. It adds that “the duration of coma was inversely correlated with the children’s IQ at 12 months after recovery” from a hyperammonaemic coma [35].

A study on paediatric hyperammonaemia lists coma duration, ammonia peak level, and hyperammonaemia duration as the main risk factors for neurological deficits and death. The authors emphasize that time critical management and a reduced duration of the hyperammonaemic coma can prevent adverse neurological outcomes [36]. A review examining the neurotoxicity of hyperammonaemia on the developing brain highlights that the extent of irreversible brain damage depends on both the magnitude and duration of the exposure to ammonia [37]. The developing brain is more susceptible to the toxic effects of ammonia. Therefore, in newborns and infants, even relatively low but prolonged exposure can cause severe and irreversible damage, such as cerebral atrophy and seizures.

It is also reported that prolonged hyperammonaemia, leading to sustained cerebral oedema, can result in permanent brain damage. The swelling of astrocytes is a key part of this pathology, and the cumulative effect of prolonged exposure can cause greater harm than a transient, high peak [38]. A high peak ammonia level is a sign of acute, severe toxicity, a longer duration of exposure causes a cumulative toxic effect on brain cells, particularly astrocytes, the cells involved in ammonia detoxification in the brain. Hyperammonaemia interferes with the metabolic function of astrocytes, leading to swelling and disruption of brain-blood barrier integrity. Prolonged exposure exacerbates this damage, causing more widespread neurological dysfunction. Cerebral oedema leads to raised intracranial pressure, which may result in brain herniation [39].

It is suggested that alterations in the neurotransmitter system are responsible for the neuronal damage seen in both acute and chronic hyperammonaemia. Animal studies show acute ammonia intoxication in vitro results in elevated extracellular levels of glutamate in the brain. The resultant activation of the N-methyl-D-aspartate (NMDA) receptors is the most probable cause of seizures in acute hyperammonaemia [40]. Patients may develop intellectual disability, behavioural and psychiatric symptoms in chronic hyperammonaemia. This has been linked to glutamine levels in the brain [41].

### Background ammonia levels

Background ammonia levels, defined as ammonia concentrations measured outside episodes of metabolic decompensation, are one of the important indicators of long-term metabolic control. Persistent reduction of ammonia levels is expected to reduce cumulative neurotoxicity and chronic energy deficiency. Haberle et al. suggested that, for the majority of the measurements obtained during the first year after diagnosis, ammonia levels taken during outpatient clinic visits were elevated in the range of 70–140 µmol/L, yet no clinical signs of acute hyperammonaemia were observed and thus no specific treatment was administered [42]. However, PROTECT data demonstrate that this low-level elevation was persistent for longer than a year indicating sustained suboptimal metabolic control.

Long-term Carbaglu^®^ treatment saw a statistically significant reduction in the mean levels of background ammonia to 51 µmol/L (28%). This reduction is clinically relevant, as background ammonia concentrations at or above 50 µmol/L have been shown to be significantly associated with impaired visual motor integration in patients with urea cycle disorders, even in the absence of acute hyperammonaemic episodes [43]. Bringing mean background ammonia levels back towards the upper limit of the normal range therefore represents not only biochemical improvement but may also confer protection against subtle yet clinically meaningful neurodevelopmental impairment. Normal ammonia levels are generally defined as 11–60 µmol/L (adult females 11–51 µmol/L; adult males 16–60 µmol/L; newborns up to 100 µmol/L and children 4 weeks to 18 years under 50 µmol/L).

These results show that without long-term Carbaglu^®^ treatment both the mean and the median levels of background ammonia were persistently above normal. Such insidious and chronic hyperammonaemia, even when not accompanied by acute symptoms, is increasingly recognised as clinically significant and may contribute to long-term neurological and developmental morbidity. Although long-term Carbaglu^®^ treatment resulted in substantial improvement, the standard deviation of the mean values (18) and the median background ammonia concentration of 50 µmol/L indicate that a proportion of patients for significant periods of time, remained above this clinically relevant threshold. Given the links to neurodevelopmental impairment, these residual elevations warrant attention.

The presented data do not allow determination of whether dietary factors, patient activity, comorbidities, intercurrent infections, clinician practice or attitudes, or other variables contributed to these persistently elevated measurements. Nevertheless, these findings underscore both the complexity of metabolic management in PA and MMA and the need for further optimisation of treatment strategies aimed not only at preventing acute decompensation but also at achieving sustained normalisation of background ammonia levels to support long-term neurodevelopmental outcomes.

### Health-service burden

Health service burden was reviewed looking at total number of emergency room visits, visits with family practitioner, visits with specialists such as paediatricians, endocrinologist, metabolic disease specialist dietitian, family counselling etc. and hospital stays and total number of inpatient days. Unsurprisingly, for diseases of this nature there was significant health resource utilisation. Prior to long-term Carbaglu^®^, patients had on average 11.9 in-patient events which incurred 52.7 in-patient days. The number of emergency room events was 7.0 per patient.

Not only are these events linked to considerable health service cost, but they inconvenience the lives of the patient and those who care for them. Each day spent with health professionals or in a clinic or ward is a day away from normal activities. Patients and their carers may need to travel long distances for appointments, will not be able to enjoy their normal working/ school/ family day. This can also limit social, intellectual, psychological and educational development. The impact therefore of a reduction in these visits is profound. It indicates the patients are better managed, the impact of the disease on their lives is reduced and that the burden on healthcare professionals and resources is reduced. Krumholz et al. highlighted that reductions in hospitalisations support the clinic financially and improves quality of life by reducing psychological disturbances caused by the trauma of hospitalization [44].

In the subset of patients on which an analysis could be performed, across all the metrics analysed, the reduction in healthcare burden was statistically significant once patients were on long-term Carbaglu^®^. These findings support the study of Alfadhel et al. who reported that when Carbaglu^®^ was administered alongside standard chronic treatment, MMA and PA patients had a significant reduction in emergency room visits compared with standard treatment [24].

The estimated per person per year economic cost of living with a rare disease, including caregiver costs, across 9 European countries is estimated at €121,900, of which 65% (€78,900) is attributed to direct medical costs [45]. This is more than two times greater than the simple average median income in those countries. The significant reduction in health system burden on long-term Carbaglu^®^ should be seen as a major benefit of treatment particularly if similar data are found when PROTECT is complete.

Additional factors may be contributing to the significantly reduced health-service burden of patients with PA and MMA once they are treated long-term with Carbaglu^®^. Over time clinicians may adapt an individual’s treatment regimen until finding an optimum, while the patient may learn to limit and manage catabolic stressor risks more successfully, both reducing the need for inpatient support.

### Patient weight and height

The physical development of patients is a marker of nutritional impact of MMA and PA. These two conditions show very similar patterns of growth impairment. Patients often have significantly reduced height-for-age Z-scores and smaller head circumferences compared to their healthy peers. However, weight-for-age and BMI-for-age tend to be in the average to higher ranges. Studies in children with PA and MMA have found patients to be overweight for their height [46].

The growth deficits are due to a combination of the underlying disease and chronic nutritional challenges. Frequent metabolic crises, triggered by illness, result in the breakdown of body protein and fat, which significantly disrupts growth. The necessary low-natural protein diet is difficult to balance, and over-restriction can impair growth particularly when linked to overuse of the emergency home-treatment regimens. Specialized precursor-free amino acid formulas, though essential, are deficient in certain amino acids like valine and isoleucine, which are critical for proper growth [47]. The severity of growth impairment often correlates with the disease subtype. Patients with more severe, vitamin B12-nonresponsive forms (mut0) of MMA tend to have worse growth outcomes than those with milder, responsive forms (mut−, cblA, and cblB) [48]. Some OA patients may have an associated growth hormone deficiency, which can be a contributing factor to poor growth and progressive renal failure in MMA can also contribute to poor growth [49].

It is interesting that patient weight increased relative to normal but there was no improvement in patient height relative to normal, once long-term Carbaglu^®^ treatment was initiated in those patients who had experienced at least 6 months of life without long-term Carbaglu^®^ treatment. Both results though were impaired by the standard of data analysed and were statistically insignificant. This suggests no clear link between Carbaglu^®^ and PA / MMA patients’ weight and height when the patient has not been treated with long-term Carbaglu^®^ from birth. We hope future PROTECT analyses, over greater time ranges, with improved data quality and quantity, will help provide further clarity on the impact of Carbaglu^®^ on OA patients’ weight and height.

### Patients treated from birth with Carbaglu^®^

Our results indicate that initiation of long-term Carbaglu^®^ treatment from birth in patients with MMA or PA is associated with clinically meaningful benefits for both growth and metabolic stability. Patients who commenced long-term Carbaglu^®^ therapy from birth experienced, on average, fewer metabolic decompensation events per year (0.95–1.34) and fewer hyperammonaemic decompensation events per year (0.52–0.76) compared with patients who experienced at least the first six months of life without long-term Carbaglu^®^ treatment. Notably, early treated patients also demonstrated improved physical growth, with mean height percentiles closer to the median (23rd to 34th percentile), suggesting a sustained developmental advantage. As follow-up continues, we will further evaluate divergence between these cohorts to determine the long-term significance of these findings. Taken together, these data support early initiation of Carbaglu^®^ from birth as a potentially important strategy to improve metabolic control and physical development in patients with PA or MMA, warranting further investigation.

## Conclusion

18 months into the 54 month PROTECT study, the data are continuing to show that long-term treatment with Carbaglu^®^ is beneficial for PA and MMA patients and the health services that support them. The treatment significantly reduces peak ammonia levels during decompensation events, background ammonia levels and patient burden on health services. Carbaglu^®^ reduces the annualised rate of all metabolic decompensation events including hyperammonaemic decompensation events. An interesting finding is the increased positive effects in a PA or MMA patient’s development when long-term Carbaglu^®^ treatment is started in early life. Analyses at future timepoints may help to further validate these findings.

In future analyses we aim to provide more information on how Carbaglu^®^ dosage and patient diet impact the metrics assessed, conduct PA specific and MMA specific analysis and further investigate the impact of long-term treatment of Carbaglu^®^ on PA and MMA patients from birth.

## Data Availability

The datasets used during the current study are available from the corresponding authors on reasonable request.
